# Introduction of ultra-high-field MR brain imaging in infants: vital parameters, temperature and comfort

**DOI:** 10.1016/j.ynirp.2023.100175

**Published:** 2023-06

**Authors:** I.M. van Ooijen, K.V. Annink, M.J.N.L. Benders, J. Dudink, T. Alderliesten, F. Groenendaal, M.L. Tataranno, M.H. Lequin, J.M. Hoogduin, F. Visser, A.J.E. Raaijmakers, D.W.J. Klomp, E.C. Wiegers, J.P. Wijnen, N.E. van der Aa

**Affiliations:** a Department of Neonatology, University Medical Center Utrecht, Utrecht Brain Center, University Utrecht, Utrecht, the Netherlands; b Centre for Image Sciences, High Field MR Research, University Medical Center Utrecht, University Utrecht, Utrecht, the Netherlands; c Departement of Radiology, Division of Imaging and Oncology, University Medical Center Utrecht, University Utrecht, Utrecht, the Netherlands; d Department of Biomedical Engineering, Eindhoven University of Technology, Eindhoven, the Netherlands

**Keywords:** Infants, Ultra-high-field (7 Tesla) MRI, Neuroimaging, Safety

## Abstract

**Background:**

Brain MRI in infants at ultra-high-field scanners might improve diagnostic quality, but safety should be evaluated first. In our previous study, we reported simulated specific absorption rates and acoustic noise data at 7 Tesla.

**Methods:**

In this study, we included twenty infants between term-equivalent age and three months of age. The infants were scanned on a 7 Tesla MRI directly after their clinically indicated 3 Tesla brain MRI scan. Vital parameters, temperature, and comfort were monitored throughout the process. Brain temperature was estimated during the MRI scans using proton MR spectroscopy.

**Results:**

We found no significant differences in vital parameters, temperature, and comfort during and after 7 Tesla MRI scans, compared to 3 Tesla MRI scans.

**Conclusions:**

These data confirm our hypothesis that scanning infants at 7 Tesla MRI appears to be safe and we identified no additional risks from scanning at 3 Tesla MRI.

## Highlights

• There was no difference in vital parameters, temperature, and comfort in infants between 3 T and 7 T MRI.
• Scanning infants at 7 T MRI appears to be safe.
• Future research should elucidate the advantages of 7T compared to 3T MRI, possibly improving diagnostic quality.

## Abbreviations

**MRI**: magnetic resonance imaging
**T**: Tesla
**SAR**: specific absorption rate
**IEC**: International Electrotechnical Commission
**CS**: COMFORT scale
**HR**: heart rate
**SpO2**: oxygen saturation
^**1**^**H-MRS**: ^1^H-magnetic resonance spectroscopy

## 1 Introduction

Magnetic resonance imaging (MRI) is an important neuro-imaging tool to assess brain injury and development in critically-ill neonates and infants. Brain MRI in neonates and infants is usually performed on 3 Tesla (T) MR systems. MRI at a stronger magnetic field, i.e. 7T, potentially increases the spatial resolution (image quality) and signal to noise ratio, and is beneficial for the use of advanced imaging, such as MR spectroscopic imaging (Dagia and Ditchfield, 2008; Van Der Kolk et al., 2013) and vascular imaging (Springer et al., 2016). Infants admitted to the neonatal intensive care unit are at risk for neurodevelopmental delays, even in the absence of overt brain injury (Chau et al., 2013). In this population, ultra-high-field MRI allows studying other biomarkers of metabolism or perfusion which may be a better predictor of outcome. In a previous paper, we showed some potential benefits for advanced MR imaging of neonates and infants at 7T, such as a higher signal-to-noise ratio to facilitate higher spatial resolution and better spectral dispersion to improve the detection of metabolites (Annink et al., 2020b).

Despite the lack of data, the United States Food & Drug Administration (FDA) noted scanning at field strengths greater than 4T a significant risk for infants aged ≤1 month, and scanning at field strengths greater than 8T a significant risk for infants aged >1 month (Center for Devices and Radiological Health, 2018). The FDA did not describe whether the infants' age should be corrected for gestational age at birth when scanning preterm infants. Since higher magnetic field strengths pose potential risks, infants' safety should be evaluated before any research studies or clinical work-up of MRI at 7T in (preterm born) neonates or infants can be conducted.

Previously, it was shown that the simulated global specific absorption rate (SAR) and peak local SAR of a virtual infant model of 8 weeks of 4.3 kg did exceed the simulated SAR levels of an adult model. However, the SAR per B1^2^ was lower in the infant model in the center position of the head coil than in the adult model, meaning that less power is needed to reach the same B1^2^ (Annink et al., 2020b). Also, Malik et al. (2021) showed higher simulated SAR levels in two neonate models compared to an adult model using a generic adult RF head coil for 7T (Malik et al., 2021). They also showed that, based on thermal simulations, changes in core temperature remained within International Electrotechnical Commission (IEC) limits when scanning infants at 7T for up to 1 h (Malik et al., 2021). Furthermore, we developed an acoustic hood that reduces the acoustic noise levels at the infants' ears to the same or lower dB levels than what is accepted for regular 3T MRI scans in neonates and infants when used in combination with regular hearing protection for infants (MiniMuffs and earplugs) (Huijing et al., 2021).

This study aimed to assess the safety of MR imaging at 7T in (pre)term born infants scanned between term (equivalent) age and three months of age. The infants' vital parameters and comfort might be influenced by peripheral nerve stimulation due to the time-varying gradients magnetic field (dB/dt), and the infants' temperature by an increase in SAR due to the time-varying RF magnetic field (B_1_) (Stafford, 2020). The vital parameters and temperature could be quantitatively measured, but assessing the comfort is more difficult in infants. Therefore, we used the COMFORT scale score, as regularly clinically used in pediatric and neonatal intensive care units to assess distress in neonates (Ambuel et al., 1992). We measured the infants’ vital parameters (heart rate, oxygen saturation, and number of adverse events (like respiratory or circulatory problems such as apnea >20 s or intubation, defined according to Plaisier et al., 2012 (Plaisier et al., 2012)), temperature (rectal, skin and brain temperature), and comfort (COMFORT scale (CS) scores, discomfort during MRI and feeding intolerance) during and directly after the 7T MRI scan and compared this to the same measurements during and directly after the 3T MRI scan.

## 2 Methods

### 2.1 Study population

We included neonates and infants with a clinical indication for brain MRI at 3T between term (equivalent) age and three months of age. We excluded infants with MR specific exclusion criteria, infants who were hemodynamically unstable, infants with respiratory support, and infants with a foreign body.

### 2.2 MRI procedures

Infants that participated in this study were first scanned at a 3T MR scanner with an 8 or 32-channel receive head coil (both Philips Medical Systems, Best, Netherlands) as part of standard clinical care. All were comfortable and hemodynamically stable after their 3T MRI scan. Directly after the 3T MRI they underwent a 7T MRI scan (Philips Medical Systems, Best, Netherlands). For the 7T MRI scan, we used a 2-channel transmit 32-channel receive head coil (Nova Medical, Wilmington, MA). More information about the sequences was published before (Annink et al., 2020b). The SAR and sound pressure levels as calculated by the scanner software were attempted to be made equivalent for 3T and 7T. The maximum duration of each MRI scan was 45 min.

According to our clinical protocol, all infants were sedated with chloral hydrate (30–50 mg/kg) via a nasogastric tube before the 3T MRI scan. Infants were placed in a vacuum pillow to prevent motion during MRI (Med Vac Infant Immobilizer Bag, Radstadt, Austria). If needed, we administered an extra feeding via the nasal feeding tube to stimulate natural sleep just before the 7T MRI scan, but no second dose of chloral hydrate was given before the 7T MRI scan.

We protected the infants' middle ears with Minimuffs (Natus Medical Incorporated, San Carlos, CA, USA), Earmuffs (EM's 4 Kids, Brisbane, Australia), and a home-built acoustic hood (Huijing et al., 2021) placed over the head coil. After the MRI scans, we monitored all infants for at least 12 h (range 12–18 h), according to standard clinical guidelines for evaluating the possible effects of sedation given prior to the 3T MRI scan.

### 2.3 Safety measures

#### 2.3.1 Safety measures - vital parameters

We continuously measured heart rate (HR) and peripheral oxygen saturation (SpO2) from 1 to 2 h before until 12–18 h after both MRI scans. Before and during both MRI scans, an MR-compatible pulse oximeter was attached to the infants’ toe (Nonin, Minneapolis, MN). After the MRI scans, we either used the same pulse oximeter or a clinical patient monitor (Philips IntelliVue MP70 Neonatal patient monitor, Philips Medical Systems, Best, Netherlands). We removed movement artifacts from the data and calculated average HR and basal SpO2 (defined as the average of the SpO2 readings not including any desaturation event) for the different periods (before 3T MRI, during 3T, during 7T, and after 7T MRI). Furthermore, we registered the number of episodes of HR > 200 bpm, HR < 100 bpm, SpO2 < 85% and apnea.

According to Plaisier et al. (2012), we monitored additional adverse events related to vital parameters (Plaisier et al., 2012). The minor adverse events are: respiratory instability resulting in increased respiratory support (need for 2–3 L of low-flow oxygen) and increased hemodynamic instability (an increase of more than five episodes of HR < 100 bpm, SpO2 < 85%, or apnea >20 s) without the need for inotropic agents. The major adverse events are: respiratory problems leading to intubation, circulatory problems leading to the need for inotropic agents, resuscitation, and death.

We contacted the parents one week after the scans to inquire about any MRI-related adverse events i.e. feeding problems.

#### 2.3.2 Safety measures - temperature

The nurses measured the rectal temperature with a standard rectal thermometer (SCALA Electronic GmbH, Stahnsdorf, Germany) before the 3T MRI scan, after the 7T MRI scan and 0–6, 6–12, and 12–18 h after both MRI scans.

During the MRI scans, we measured the skin temperature via an MR-compatible temperature sensor (Neoptic Reflex Fiber Optic Temperature Sensor, Quebec, Canada) attached to the abdomen at the level of the liver. The physician (assistant) monitored the skin temperature during both MRI scans, whereby hypothermia was defined as a temperature <35.5 °C and hyperthermia was defined as a temperature >38.5 °C.

We estimated the brain temperature using ^1^H-magnetic resonance spectroscopy (^1^H-MRS) data (3T: PRESS, TE/TR = 38/2000ms; 7T: STEAM, TE/TR = 10/2000ms or sLASER, TE/TR = 36/2000ms) during both MRI scans, from a single voxel in the left deep gray matter. Two independent MRS experts (JW and EW) checked the MRS data quality on a scale of three: good, moderate and poor. We used the data if both experts scored the scan quality as good or moderate. The STEAM sequence was preferably used, so only in cases of a poor-quality STEAM, we used the sLASER sequence. Based on these scans, we estimated the brain temperature by assessing the chemical shift difference between the water peak and the N-acetyl-aspartate (NAA) peak (ΔH2O−NAA) in parts per million (ppm) (Alderliesten et al., 2017b; Annink et al., 2020a; Wu et al., 2014). We used the water-suppressed scan to determine the frequency position of the NAA peak and the (non-suppressed) water scan to determine the frequency position of the water peak. Frequency positions were determined by AMARES peak fitting in jMRUI (Vanhamme et al., 1997). Brain temperature (T) was calculated as *T* = 263 − (ΔH2O − NAA × 85.76) (Annink et al., 2020a).

#### 2.3.3 Safety measures - comfort

A trained physician (assistant) or nurse assessed the COMFORT scale score (Ambuel et al., 1992) at admission, during transport to the 3T MRI, between the MRI scans, after the 7T MRI scan, and 0–6, 6–12 and 12–18 h after the 7T MRI. The COMFORT scale is a scoring system containing six items with a 5-point scale to score the comfort of the baby (1 = comfortable and 5 = discomfort). The scored items are alertness, agitation, crying, body movements, muscle tension in the face, and muscle tension in the body. A score of 14 or more indicates the baby experiences discomfort.

In addition to the COMFORT scale, we recorded the need for the interruption and prematurely stopping of the MRI scans because of possible discomfort (crying, inadequate monitoring due to movement or movement artifacts on the scan) or feeding intolerance.

### 2.4 Statistics

We compared differences between values measured during 3T and 7T MRI (average HR, basal SpO2, and brain temperature). In case of no measurements during the MRI scans, we compared before and after both MRI scans (rectal temperature), and right after the 3T and 7T MRI scan (CS scores). We compared all these differences using a paired-samples *t*-test (normally distributed data) or a Wilcoxon signed ranks test (not normally distributed data), using SPSS Statistics version 27 (SPSS Inc. Released, 2008). To calculate the probability of the null hypothesis (no difference between 3T and 7T) being true, we used the Bayesian paired samples *t*-test with the Bayes factor in favor of the null hypothesis (BF_01_), using the JASP statistical software package version 17.1 (JASP Team, 2023).

### 2.5 Ethical approval

All parents gave written informed consent. The medical ethical committee of the University Medical Centre Utrecht approved this study (NL66198.041.18). This work has been carried out in accordance with The Code of Ethics of the World Medical Association (Declaration of Helsinki).

## 3 Results

### 3.1 Study population

Between January 2019 and August 2021, we included twenty neonates and infants in this study. Table 1 shows the baseline characteristics. Eleven infants were born (extremely) preterm and had a clinical indication for an MRI scan at term equivalent age. Nine were born at term and had a clinical indication for an MRI scan between birth and three months of age. The weight of the neonates and infants during MRI ranged between 2715 and 6700 g. One patient could not be scanned at 7T MRI, due to technical problems with the temperature sensor. These data were excluded in the analyses.

**Table 1:** Baseline characteristics of the infants included in this study.

Characteristic	Infants (n = 20)
Sex (male), *n (%)*	11 (55)
Gestational age at birth (wk), *mean (sd)*	33.1 (1.5)
Birthweight (g), *mean (sd) [range]*	2107 (1376) [585–4570]
Apgar score 5 min, *median (range)*	8 (2–10)
Apgar score 10 min, *median (range)*	9 (6–10)
Post menstrual age MRI (wk), *mean (sd)*	44.6 (1.1)
Postnatal days MRI, *mean (sd)*	80.5 (7.1)
Weight at MRI (g), *mean (sd) [range]*	4273 (1313) [2715–6700]
Indication MRI, *n (%)*	
o Extremely preterm birth (<28 weeks of gestation)	9 (45)
o Follow-up MRI brain hemorrhage	2 (10)
o Follow-up MRI brain ischemia	5 (25)
o Follow-up MRI brain cyst	1 (5)
o Follow-up MRI perinatal asphyxia without hypothermia	1 (5)
o Cerebral sinovenous thrombosis (CSVT)	1 (5)
o Metabolic disorder	1 (5)

### 3.2 Safety measures

#### 3.2.1 Safety measures - vital parameters

**Fig. 1:**
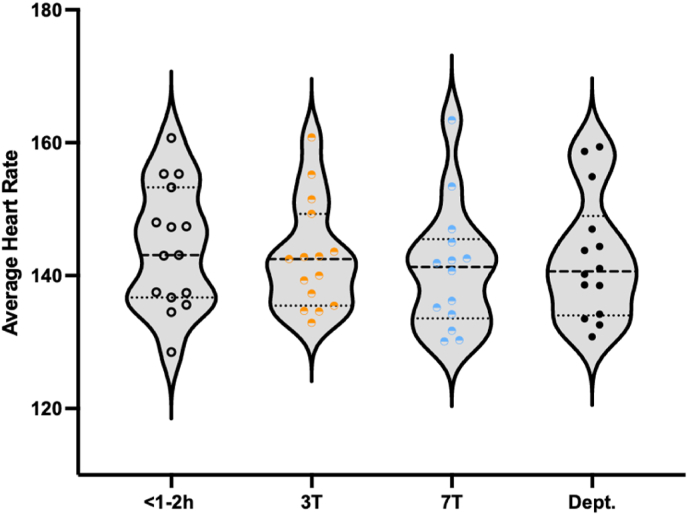
Average heart rate (HR) measured from start until 3T (o), during 3T (), during 7T () and from the transport to the department (Dept.) until the end of monitoring (●).

**Fig. 2:**
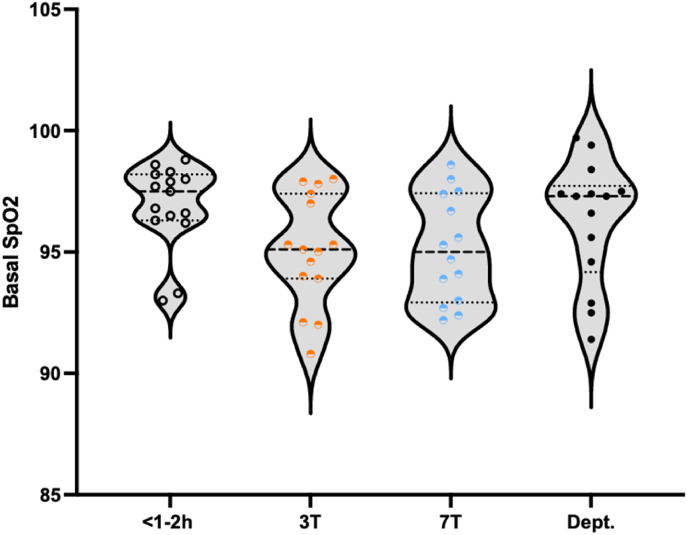
Basal SpO2 (steady-state SpO2: average of the SpO2 readings that are not included in any desaturation event) measured from start until 3T (o), during 3T (), during 7T () and from the transport to the department (Dept.) until the end of monitoring (●).

The average HR (Fig. 1) and Basal SpO2 (Fig. 2) did not differ between 3T and 7T (mean average HR: 143 and 141 respectively; *Z =* -0.772; *p =* 0.470, supported by a BF_01_ (Bayes factor in favor of the null hypothesis) of 2.090 (0.016% error); and mean basal SpO2: 95.1 and 95.1, respectively; Z = −0.050; p = 0.294, supported by a BF_01_ of 2.614 (0.013% error)). Out of the nineteen patients, four patients had missing HR and SpO2 data due to failure in saving the continuous data, and one patient had unreliable data due to leg movement during and after the 7T scans. In these infants, we averaged the HR and SpO2 values written down at the start and the end of the MRI scans. These were all within normal ranges (average HR at 3T: 130; 158; 139 and 148 bpm and average HR at 7T: 130; 108; 140; 128 and 147 bpm, for each of these patients respectively. Average SpO2 at 3T: 96; 95; 92 and 97% and average SpO2 at 7T: 98; 96; 95; 95 and 95%, for each of these patients respectively).

As shown in Table 2, SpO2 < 85% occurred in one infant with spontaneous recovery (before arrival in the hospital, during 3T, during 7T, and at the department after both MRI scans), born extremely preterm. This infant was known for this problem and already received daily caffeine administrations. A HR > 200 beats per minute occurred in 3 infants: in one infant before the 3T MRI scan, in one infant during the 7T MRI scan, and in one infant after the 7T MRI scan. All three periods were short and induced by crying since the HR directly recovered after comforting the infant. None of the infants experienced a HR < 100 bpm or apnea.

**Table 2:** All observed adverse parameters and signs of discomfort throughout the study.

Event	Before 3T (n)	During 3T (n)	During 7T (n)	After 7T (n)
**Oxygen saturation (SpO**_**2**_**) < 85%**	1^	1	1	1
**Heart rate (HR) > 200 bpm**	1	0	1	1
**Regurgitation of milk**	11	1	0	1
**Interruption of scanning**	–	5 (3xa, 1xb, 1xc^a^)	5 (4xa, 1xc^a^)	–
**Scan stopped prematurely**	–	0	1 (1xd^a^)	–

a a: crying but able to comfort; b: inadequate monitoring (saturation monitor disconnected); c: movement artifacts scan; d: technical problems (temperature sensor not working). ^At home, before arrival on department.

According to the definition of Plaisier et al. (2012), there were no minor or major adverse events before, during, or after the MRI scans (Plaisier et al., 2012). Also, the parents did not report any MRI-related adverse events in the week after the MRI scans.

#### 3.2.2 Safety measures - temperature

**Fig. 3:**
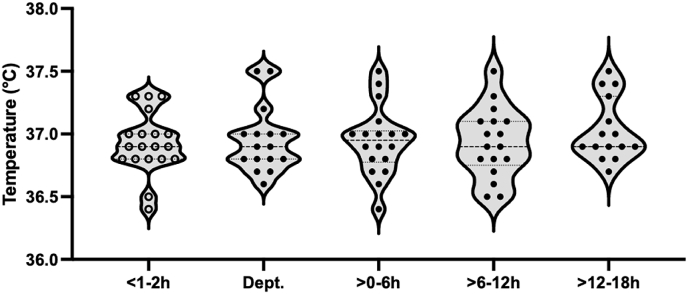
The rectal temperature is depicted 1–2 h before MRI (o), after return on department (Dept.), and 0–6, 6–12, and 12–18 h after both MRI scans (●).

**Fig. 4:**
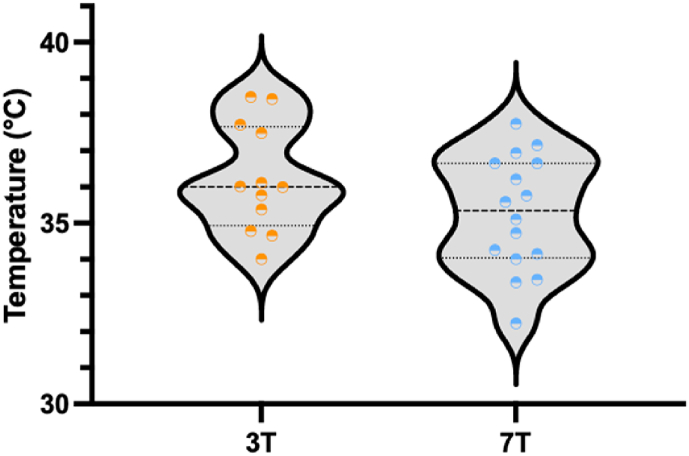
The brain temperature is estimated during 3T () and 7T () MRI, using ^1^H-MRS in the left deep gray matter.

Rectal temperature was stable (Fig. 3) before and after both MRI scans (mean rectal temperature: 36.9 °C and 36.9 °C at 3T and 7T, respectively; *Z =* -0.286; *p =* 0.775, supported by a BF_01_ of 3.363 (0.013% error)). In 10 out of 95 measurements, rectal temperature data was missing. Rectal and skin temperature during the MRI scans showed no periods of hypothermia (<35.5 °C) or hyperthermia (>38.5 °C). Also, brain temperature did not differ between 3T and 7T MRI (mean temperature: 36,2 °C and 35,3 °C, respectively; *Z =* -1.070; *p =* 0.285, supported by a BF_01_ of 2.273 (0.010% error)) (Fig. 4). In 10 out of 38 measurements, MRS-derived brain temperature data was missing (seven cases of missing data (six at 3T and one at 7T) and three cases of insufficient quality (one at 3T and two at 7T)).

#### 3.2.3 Safety measures - comfort

**Fig. 5:**
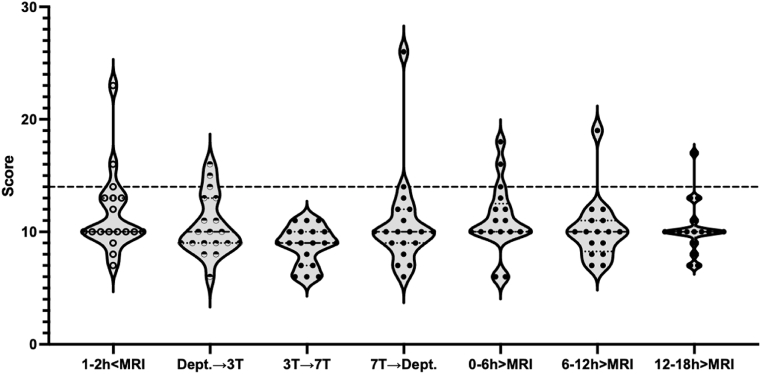
COMFORT scale (Ambuel et al., 1992) scores measured 1–2 h before MRI, during transport between MRI scans, and every 6 h after the MRI scans until 18 h after MRI scans. A score of 14 or more means the baby experiences discomfort, as indicated by the dotted line. *Dept*. *= department; → = transport*. ≤ *before;* ≥ *after*.

A higher CS score means less comfort, whereby a CS score above 14 indicates discomfort. CS scores were not statistically different after 3T MRI compared to after 7T MRI (mean CS: 8.8 and 10.7 after 3T and 7T, respectively; *Z =* -1.851; *p =* 0.064) (Fig. 5), not clearly supported by a BF_01_ of 0.988 (0.024% error)). In 8 out of 133 measurements, the CS scores were missing. A CS score of 14 or higher (discomfort) was seen 1–2h before the MRI scans (n = 3; CS = 14/16/23), during transport to the 3T MRI (n = 3; CS = 14/15/16), during transport after the 7T MRI scan (n = 2; CS = 14/26), and 0–6 h (n = 3; CS = 14/16/18), 6–12 h (n = 1; CS = 19) and 12–18 h (n = 1; CS = 17) after both MRI scans. One infant showed a highly increased CS score (CS = 23) before both MRI scans and another infant showed a highly increased CS score (CS = 26) during transport after the 7T MRI. Both highly increased CS scores were most likely due to hunger since the CS score quickly recovered after feeding the infants. The other eleven CS scores of 14 or higher (discomfort) were between 14 and 19, all CS scores quickly recovered.

The 3T and 7T MRI scans were both paused five times (Table 2). In three infants at 3T and in four infants at 7T because of crying (all were easily comforted), in one infant at 3T and 7T because of movement artifacts, and in one infant at 3T because of inadequate monitoring (the saturation monitor was disconnected). Eleven infants showed regurgitation of milk before both MRI scans. One infant showed regurgitation of milk during the 3T MRI scan, which already occurred at the department before both MRI scans. One infant showed regurgitation of milk after the 7T MRI scan, which already occurred at the department before both MRI scans.

## 4 Discussion

In our previous paper, we measured sound pressure levels and modeled SAR levels of neonatal MRI at 7T (Annink et al., 2020b). This paper assessed the other possible effects of the higher RF at 7T when compared to 3T (298 MHz versus 128 MHz, respectively) and higher static magnetic field (7T versus 3T) by monitoring the infants’ vital parameters, temperature, and comfort. Vital parameters, temperature, and comfort in infants were not statistically different comparing the 7T MRI scan to the 3T MRI scan.

There were no significant differences in vital parameters (mean HR and basal SpO2) when comparing the 3T MRI scan to the 7T MRI scan, supported by the bayes factor. However, basal SpO2 was lower during both MRI scans when compared to before and after scanning (during transport and at the department). This could be a possible effect of being asleep after sedation or because of the use of the acoustic hood, but it is beyond the scope of this research. The saturation stayed within a safe range, so no intervention was necessary. Since lower SpO2 appeared during both scans, and we studied safety of 7T MRI compared to 3T we concluded this was not a side effect of high field strength. Also, we found no increase in episodes of HR > 200 or SpO2 < 85%, no episodes of HR < 100 or apnea, and no AE's. Therefore, the 7T MRI scan shows no additional risk on vital parameters as compared to the 3T MRI scan.

There were no significant differences in rectal temperature before 3T and after 7T MRI, supported by the bayes factor. There were no cases of hypo- or hyperthermia for both rectal and skin temperature. There was no significant difference in brain temperature between 3T and 7T. Brain temperature is presented as an absolute value but could be aberrant from real brain temperature as this depends on the accuracy of the calibration curve that was used. Nonetheless, since the B0 field is not a parameter in determining the brain temperature, a deviation of real temperature would be equal for 3T and 7T (De Graaf, 2013). The brain temperature is comparable to the observed brain temperatures in previous studies (Annink et al., 2020a; Wu et al., 2014). Therefore, the 7T MRI scan shows no additional risks on temperature as compared to the 3T MRI scan.

There were no significant differences in CS scores after a 3T MRI compared to after a 7T MRI procedure, although not supported by the bayes factor. In 13 out of 125 measurements (8 were missing), the infant had a COMFORT scale score above 14. A COMFORT scale of 14 and above indicates the baby experiences discomfort. Only two of those 13 cases, happened directly after the 7T MRI. In one of those (CS = 26), the discomfort was explained by hunger as the baby was comforted easily upon feeding. The other had a comfort score of 14 and the infant quickly recovered upon return to the department. The bayes factor <1.0 is driven by this high CS score of 26. Also, interruption of scanning and feeding intolerance were not increased during or after the 7T MRI scan. Therefore, the 7T MRI scan shows no additional risks on comfort as compared to the 3T MRI scan.

There are some limitations to this study. First, we did not randomize the order of the 3T and 7T MRI. Randomization was not possible due to the clinical importance of the 3T MRI scan results. As a result, sedation was given only before the 3T MRI and could have already worn off in some infants during the 7T MRI, leading to a potential bias of discomfort during the 7T MRI (albeit not observed). Second, because of our inclusion criteria, these results do not apply to hemodynamically unstable infants (i.e., respiratory support) or infants with a foreign body (i.e., intravenous catheter). Third, we attempted to make the 3T and 7T MRI protocols equivalent in terms of SAR and sound pressure deposition. However, due to intrinsic differences between scanners and hardware (for example, bore coil excitation at 3T, versus local excitation at 7T with a head coil) it is deemed impossible to have exactly similar scan conditions. Of note, it is advised to review SAR simulations at 7T when a different transmit coil is used. Finally, vital parameters had some missing continuous data but were verified with non-continuous data written down during the process.

Future research should elucidate the advantages of 7T MRI compared to 3T MRI, possibly improving diagnostic quality in neonates and infants. Preliminary results already suggest image quality improvements at 7T MRI compared to 3T MRI, especially for vascular imaging: magnetic resonance angiography (MRA), susceptibility-weighted imaging (SWI), phase-contrast magnetic resonance imaging (PC-MRI) and metabolic maturation: magnetic resonance spectroscopy (MRS) (Annink et al., 2020b).

## 5 Conclusion

In this paper, we demonstrated that 7T MR scanning in stable neonates and infants (from term (equivalent) age up to three months of age) induces no significant changes in vital parameters, temperature, and comfort compared to 3T MRI. Therefore, 7T MRI appears to be safe in stable infants without respiratory support and there are no additional risks when compared to 3T MRI.

## CRediT authorship contribution statement

**I.M. van Ooijen:** Data curation, Formal analysis, Investigation, Project administration, Visualization, Writing – original draft. **K.V. Annink:** Conceptualization, Data curation, Investigation, Methodology, Project administration, Writing – review & editing. **J. Dudink:** Investigation, Writing – review & editing. **T. Alderliesten:** Investigation, Writing – review & editing. **F. Groenendaal:** Conceptualization, Investigation, Writing – review & editing. **M.L. Tataranno:** Investigation, Writing – review & editing. **M.H. Lequin**: Writing – review & editing. **J.M. Hoogduin:** Conceptualization, Writing – review & editing. **F. Visser:** Conceptualization, Investigation, Writing – review & editing. **A.J.E. Raaijmakers:** Conceptualization, Writing – review & editing. **D.W.J. Klomp:** Supervision, Writing – review & editing. **E.C. Wiegers:** Supervision, Investigation, Writing – review & editing. **M.J.N.L. Benders:** Conceptualization, Supervision, Writing – review & editing. **J.P. Wijnen:** Conceptualization, Funding acquisition, Investigation, Supervision, Writing – review & editing. **N.E. van der Aa:** Conceptualization, Funding acquisition, Investigation, Supervision, Writing – review & editing.

## Funding

This work was supported by a grant from the Dr. C.J. Vaillant foundation (no number) and the WKZ onderzoeksfonds (WKZ210407Wiegers).

## Declaration of competing interest

The authors declare the following financial interests/personal relationships which may be considered as potential competing interests:

F. Visser has a partial employment with a commercial organization (Philips Healthcare, Best, The Netherlands) and F. Groenendaal is an expert witness in medicolegal cases. The other authors report no disclosures.

## Data Availability

The *clinical data* in this study is not available upon request. There is no approval from the medical ethical committee of the University Medical Centre Utrecht since the data incorporates privacy issues. The *MRI data* is available for collaboration with other research teams on receipt of a reasonable request to access the study data. Requests can be sent to the corresponding author. If the request is found appropriate, the corresponding author will share the pseudonymized MRI data.
